# A Hybrid Architecture (CO-CONNECT) to Facilitate Rapid Discovery and Access to Data Across the United Kingdom in Response to the COVID-19 Pandemic: Development Study

**DOI:** 10.2196/40035

**Published:** 2022-12-27

**Authors:** Emily Jefferson, Christian Cole, Shahzad Mumtaz, Samuel Cox, Thomas Charles Giles, Sam Adejumo, Esmond Urwin, Daniel Lea, Calum Macdonald, Joseph Best, Erum Masood, Gordon Milligan, Jenny Johnston, Scott Horban, Ipek Birced, Christopher Hall, Aaron S Jackson, Clare Collins, Sam Rising, Charlotte Dodsley, Jill Hampton, Andrew Hadfield, Roberto Santos, Simon Tarr, Vasiliki Panagi, Joseph Lavagna, Tracy Jackson, Antony Chuter, Jillian Beggs, Magdalena Martinez-Queipo, Helen Ward, Julie von Ziegenweidt, Frances Burns, Joanne Martin, Neil Sebire, Carole Morris, Declan Bradley, Rob Baxter, Anni Ahonen-Bishopp, Paul Smith, Amelia Shoemark, Ana M Valdes, Benjamin Ollivere, Charlotte Manisty, David Eyre, Stephanie Gallant, George Joy, Andrew McAuley, David Connell, Kate Northstone, Katie Jeffery, Emanuele Di Angelantonio, Amy McMahon, Mat Walker, Malcolm Gracie Semple, Jessica Mai Sims, Emma Lawrence, Bethan Davies, John Kenneth Baillie, Ming Tang, Gary Leeming, Linda Power, Thomas Breeze, Duncan Murray, Chris Orton, Iain Pierce, Ian Hall, Shamez Ladhani, Natalie Gillson, Matthew Whitaker, Laura Shallcross, David Seymour, Susheel Varma, Gerry Reilly, Andrew Morris, Susan Hopkins, Aziz Sheikh, Philip Quinlan

**Affiliations:** 1 Health Informatics Centre Division of Population and Health Genomics School of Medicine, University of Dundee Dundee United Kingdom; 2 Digital Research Service University of Nottingham Nottingham United Kingdom; 3 Usher Institute University of Edinburgh Edinburgh United Kingdom; 4 Health Data Research UK London United Kingdom; 5 Lay Partnership in Healthcare Research Lindfield United Kingdom; 6 National Health Service Digital London United Kingdom; 7 School of Public Health Imperial College London London United Kingdom; 8 Department of Haemotology University of Cambridge Cambridge United Kingdom; 9 National Institute for Healthcare Research BioResource Cambridge University Hospitals NHS Foundation Cambridge Biomedical Campus Cambridge United Kingdom; 10 Centre for Public Health Belfast Institute of Clinical Science Queens University Belfast Belfast United Kingdom; 11 Blizard Institute Faculty of Medicine and Dentistry Queen Mary University of London London United Kingdom; 12 Institute of Child Health Great Ormond Street Hospital London United Kingdom; 13 Public Health Scotland Edinburgh United Kingdom; 14 Centre for Public Health Institute of Clinical Science Queen's University Belfast Belfast United Kingdom; 15 Public Health Agency Belfast United Kingdom; 16 EPCC University of Edinburgh Edinburgh United Kingdom; 17 BC Platforms Espoo Finland; 18 Molecular and Clinical Medicine School of Medicine University of Dundee Dundee United Kingdom; 19 School of Medicine University of Nottingham Nottingham United Kingdom; 20 Institute of Cardiovascular Sciences University of College London London United Kingdom; 21 Big Data Institute University of Oxford Oxford United Kingdom; 22 Barts Heart Centre London United Kingdom; 23 Clinical and Protecting Health Directorate Public Health Scotland Glasgow United Kingdom; 24 School of Medicine University of Dundee Dundee United Kingdom; 25 Population Health Sciences Avon Longitudinal Study of Parents and Children Bristol United Kingdom; 26 Radcliffe Department of Medicine Oxford University Oxford United Kingdom; 27 Oxford University Hospitals NHS Foundation Trust John Radcliffe Hospital Oxford United Kingdom; 28 British Heart Foundation Cardiovascular Epidemiology Unit Department of Public Health and Primary Care University of Cambridge Cambridge United Kingdom; 29 British Heart Foundation Centre of Research Excellence University of Cambridge Cambridge United Kingdom; 30 National Institute for Health Research Blood and Transplant Research Unit in Donor Health and Behaviour University of Cambridge Cambridge United Kingdom; 31 Health Data Research UK Cambridge Wellcome Genome Campus University of Cambridge Cambridge United Kingdom; 32 Health Data Science Research Centre Human Technopole Milan Italy; 33 Health Protection Research Unit in Emerging and Zoonotic Infections Institute of Infections University of Liverpool Liverpool United Kingdom; 34 Respiratory Department Alder Hey Children's Hospital Liverpool United Kingdom; 35 University College London London United Kingdom; 36 BioIndustry Association London United Kingdom; 37 Outbreak Data Analysis Platform University of Edinburgh Edinburgh United Kingdom; 38 NHS England Worcestershire United Kingdom; 39 Civic Data Cooperative Digital Innovation Facility University of Liverpool Liverpool United Kingdom; 40 Public Health England London United Kingdom; 41 Avon Longitudinal Study of Parents and Children Bristol Medical School University of Bristol Bristol United Kingdom; 42 University of Birmingham Birmingham United Kingdom; 43 University Hospital Coventry & Warwickshire NHS Trust Coventry United Kingdom; 44 Population Data Science Swansea University Medical School Swansea United Kingdom; 45 Barts Heart Centre St Bartholomew's Hospital Barts Health NHS Trust London United Kingdom; 46 Institute of Cardiovascular Science University College London London United Kingdom; 47 Nottingham Biomedical Research Centre School of Medicine University of Nottingham Nottingham United Kingdom; 48 Immunisation and Countermeasures Division Public Health England Colindale London United Kingdom; 49 UK Health Security Agency London United Kingdom; 50 Institute of Health Informatics UCL London United Kingdom; 51 Centre for Population Health Sciences Usher Institute University of Edinburgh Edinburgh United Kingdom

**Keywords:** COVID-19, clinical care, public health, infrastructure model, health data, meta-analysis, federated network, health care record, data extraction, data privacy, data governance, health care

## Abstract

**Background:**

COVID-19 data have been generated across the United Kingdom as a by-product of clinical care and public health provision, as well as numerous bespoke and repurposed research endeavors. Analysis of these data has underpinned the United Kingdom’s response to the pandemic, and informed public health policies and clinical guidelines. However, these data are held by different organizations, and this fragmented landscape has presented challenges for public health agencies and researchers as they struggle to find relevant data to access and interrogate the data they need to inform the pandemic response at pace.

**Objective:**

We aimed to transform UK COVID-19 diagnostic data sets to be findable, accessible, interoperable, and reusable (FAIR).

**Methods:**

A federated infrastructure model (COVID - Curated and Open Analysis and Research Platform [CO-CONNECT]) was rapidly built to enable the automated and reproducible mapping of health data partners’ pseudonymized data to the Observational Medical Outcomes Partnership Common Data Model without the need for any data to leave the data controllers’ secure environments, and to support federated cohort discovery queries and meta-analysis.

**Results:**

A total of 56 data sets from 19 organizations are being connected to the federated network. The data include research cohorts and COVID-19 data collected through routine health care provision linked to longitudinal health care records and demographics. The infrastructure is live, supporting aggregate-level querying of data across the United Kingdom.

**Conclusions:**

CO-CONNECT was developed by a multidisciplinary team. It enables rapid COVID-19 data discovery and instantaneous meta-analysis across data sources, and it is researching streamlined data extraction for use in a Trusted Research Environment for research and public health analysis. CO-CONNECT has the potential to make UK health data more interconnected and better able to answer national-level research questions while maintaining patient confidentiality and local governance procedures.

## Introduction

COVID-19 introduced a new set of conditions to existing challenges in health and clinical data collection within the United Kingdom. Regularly updated data were required at pace to inform decision-making and research, but were being generated by heterogenous sources, such as new “Lighthouse” laboratories [1] set up specifically for the pandemic, academic research laboratories, and usual primary and secondary care settings [2]. The diversity of data sources and the lack of awareness of them made it challenging to identify and access these data sources, as was highlighted by the UK Government Chief Scientific Adviser [3]. In our experience, it was often the case that each research or public sector group had to contact each potential data source individually to obtain information about the data they host, making the process complex and lengthy even for high-level questions, such as simply finding out what data are available. Such challenges are described in detail in the Goldacre Review [4] and across many studies [5-8].

Typically, any analysis of patient data or electronic health records (EHRs) requires many steps covering legal (eg, General Data Protection Regulations [GDPR] compliance) [9], operational (eg, data sharing agreements) [10,11], and security aspects (eg, access to unconsented pseudonymized or anonymized data in a secure environment where the data cannot be exported, ie, a Trusted Research Environment [TRE] [12]) [13]. These steps are crucial to ensure appropriate reuse of data but can take many months to complete before any data analysis can take place [14].

The need for more streamlined and efficient methods for discovering and analyzing EHRs is not new [15], but the COVID-19 pandemic has played a catalytic role in highlighting the need for these methods more than ever before. Data are federated when held at different locations and often hosted by different data controllers. The World Economic Forum has recently published a guideline document that focuses on sharing of sensitive health data in a federated consortium model considering the post–COVID-19 world [16]. Large-scale projects, such as the Global Alliance for Genomics and Health [17]; Canadian Distributed Infrastructure for Genomics [18]; Common Infrastructure for National Cohorts in Europe, Canada, and Africa [19]; and European Health Data and Evidence Network [20] projects, have laid out principles and frameworks supporting safe use of patient data [17,21]. While federated academic tooling (software that works on federated data sets) exists [22-25], the commercial sector appears to have more capability than the best in academia [26-28]. However, commercial systems usually come with contracts and licensing terms that may not be suitable for everyone and also focus on finding patients for recruitment to clinical trials rather than cohort discovery and meta-analysis from EHR data. Equally, the commercial nature of the systems means they are usually based on proprietary standards, which results in further fragmentation and lack of accessibility of data sets.

Given the need for more impactful solutions in accessing aggregated health data, accelerated by the pandemic [29], the COVID - Curated and Open Analysis and Research Platform (CO-CONNECT) was established at scale and at pace. The Health Data Research (HDR) Innovation Gateway [30] (Gateway) is a web resource enabling discovery of and accessibility to UK health research data, and supporting health data research in a safe and efficient manner. The Gateway provides detailed metadata descriptions of over 700 data sets held by members of the UK Health Data Research Alliance, including the Health Data Research Hubs [31]. CO-CONNECT enhances the capabilities of the Gateway by providing a query engine (the Cohort Discovery Tool) to support dynamic cohort building and meta-analysis across individual-level data from multiple data partners.

The aim of CO-CONNECT is to transform the way public health organizations and researchers discover and access COVID-19 data and associated longitudinal health care data from across the United Kingdom. This paper describes how CO-CONNECT maintains patient confidentiality and data security while supporting access to data for research at pace, and how a multidisciplinary team tackled the architecture of this platform as an asset for public health in the United Kingdom.

## Methods

### Project Initiation and Governance

CO-CONNECT was conceived early after the start of the pandemic when both researchers and public sector bodies were frantically trying to find what data existed across different data custodians to answer pressing questions, which would then inform public policy. Many research studies were being rapidly commissioned, and data were being collected via routine health care, but there was no easy way for different funders and research groups to understand what data were being collected. Once data sets had been identified, it took significant time to set up the agreements for data sharing and access.

For example, a key question at the time was whether someone would be immune to COVID-19 after contracting the disease, and if so, for how long. Low-level detailed serology results, rather than simply “positive/negative” results, were required for calibration of assays and to understand antibody responses related to individual levels of immunity. However, it was challenging for researchers to find which data controllers may be capturing low-level data, and if so, how to rapidly access the data for analysis.

These challenges where widely recognized at the time. When answering questions on the lessons to be learnt from the pandemic at the Science and Technology Committee meeting in July 2020 [3], Sir Patrick Vallance stressed the importance of data flows and data systems to support the pandemic response:

One lesson that is very important to learn from this pandemic, and for emergencies in general, is that data flows and data systems are incredibly important. You need the information in order to be able to make the decisions. Therefore, for any emergency situation those data systems need to be in place up front to be able to give the information to make the analysis and make the decisions.

The CO-CONNECT leads reached out to 26 individuals/organizations who were collecting research cohorts of data or collecting data as part of routine health care provision from the 4 devolved UK Nations to join the project as collaborators. The benefits of the platform, how it would protect patient confidentiality, and how individual-level data would not have to leave the control of the data partner needed to be rapidly communicated for each data partner to agree to the collaboration. There were 4 co-leads on CO-CONNECT, who each bought different expertise to the project and could share the duties of leading such a large project delivered within a tight timeframe during the COVID pandemic.

CO-CONNECT partnered with the National Core Studies program [32] and reported to the UK Scientific Advisory Group for Emergencies [33] through this program. The Advisory Steering Committee meets every 3 months with representatives from the 4th Pillar Testing Programme and the UK Joint Biosecurity Centre, a Chief Scientific advisor, an ethics expert, and the funders.

### Architecture of CO-CONNECT Infrastructure

#### Overview

CO-CONNECT delivers a federated capability that enables the discovery of data across multiple sources, referred to as CO-CONNECT data partners, to make them findable, accessible, interoperable, and reusable (FAIR) [34]. The federation has been designed to ensure that data can be processed in line with the GDPR and common law confidentiality requirements.

**Figure 1:**
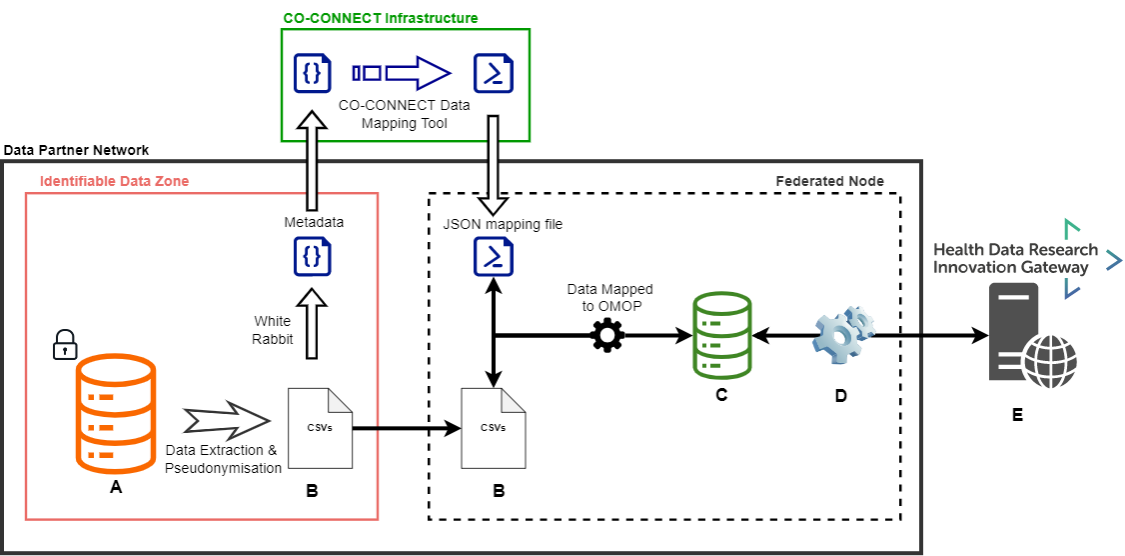
The CO-CONNECT federated architecture. A data partner (dark box) has potentially identifiable data (A) from which an extraction is made and pseudonymized (B). A metadata extraction is performed with WhiteRabbit (within the identifiable Data Zone, red box) and sent to the CO-CONNECT infrastructure (green box). A mapping script to the OMOP CDM is created using the CO-CONNECT data mapping tool (CaRROT-Mapper). The pseudonymized data are securely transferred (B) into a secure virtual machine hosted by the data partner (Federated Node, dashed dark box), mapped to OMOP (CaRROT-CDM), and connected to the federation software (C and D). From there, the data are queryable by the Innovation Gateway (E). Only aggregated fully anonymous data discovery and meta-analysis results are returned to the Gateway (D). CDM: Common Data Model; OMOP: Observational Medical Outcomes Partnership.

Figure 1 provides an architecture overview of the components that reside within the secure environment of each data partner’s network, with no inbound connectivity, and those that are available externally to researchers and the CO-CONNECT team via a secure login. Throughout the methods section, we reference the components as labeled in Figure 1 (Components A-E) in brackets after the description. Our overview video explains how the system works [35].

In summary, a secure virtual machine (VM) (Federated Node, dashed black box) is set up by the data partner, which is separate from the location where identifiable data are stored (Identifiable Data Zone, red box), but still part of their secure infrastructure. The data partner sends metadata (“Metadata” within the red box) about the data they hold to the CO-CONNECT technical team that determines the rules to map the data into the Observational Medical Outcomes Partnership (OMOP) [36] data standard format (CO-CONNECT Infrastructure, green box). The mapping script (JSON mapping file), developed by the CO-CONNECT technical team, is sent to the data partners who then apply the mapping rules to a pseudonymized version of their data (Data Mapped to OMOP). This generates a database of relevant linked and pseudonymized data sets in OMOP format within their VM (Component C, green database).

Software is installed within the VM, called BC|LINK (Component D), which provides access to the pseudonymized OMOP database (Component C, green database) and is configured to communicate with the Gateway tool (Component E) where approved users can submit queries. The Gateway contains the BC|RQUEST software (Component E) that stores the user-submitted queries and allows the BC|LINK software (Component D) to download these queries and run them against the OMOP database. Only aggregate counts are posted in response and displayed to the user. This is simultaneously repeated across all UK-wide data partners within the federation, which enables users to perform feasibility analysis (to discover relevant data from different sources) and carry out aggregate-level analysis across different UK data partners through one system.

#### Detailed Components of the Architecture

##### CaRROT Software

All CO-CONNECT developed tools (termed CaRROT [Convenient and Reusable Rapid Ontology Transformer]) are open source and freely available [37,38]. This suite of tools automates the mapping of the data into OMOP and the loading of the data into a database for external querying.

##### Access to Individual-Level Data

All individual-level data remain under the control of the data partner, and there is no requirement for any direct interaction from the CO-CONNECT pipeline with the data partner’s data systems (Database A). The federated node (dashed black box) is established on a VM that is separate from any systems that hold identifiable data.

##### ID Management and Data Linkage

All patient identifiable data are pseudonymized locally by data controllers (Data Extraction and Pseudonymization) through (1) obfuscation of potentially sensitive information, such as date of birth, and (2) removal of personal identifiable information, such as given names and addresses.

##### Generating Metadata

WhiteRabbit, from Observational Health Data Science and Informatics (OHDSI) [39], is a software tool to profile data sets to generate metadata that includes descriptions on tables, fields, and the distribution of values within each field [40]. WhiteRabbit resides within the Identifiable Data Zone but is only ever run against a pseudonymized extract of the data in CSV format (Files B), from which the WhiteRabbit report is generated. The data partner always retains control over what data WhiteRabbit can access, the configuration of the parameters, and what is shared to the CO-CONNECT team.

##### Data Mapping Tool

To ensure consistency of data across the data partners, all of the data sets are on-boarded using OMOP Common Data Model (CDM) version 5.3 [36] developed as part of the OHDSI.

We developed a data mapping tool (CaRROT-Mapper [37]; CO-CONNECT Infrastructure, green box), which ingests WhiteRabbit reports and enables the data team to generate a mapping rule to replace each field or field value to a standard OMOP vocabulary concept ID. From this concept ID, the domain can be established, which in turn confirms which table in the OMOP CDM should be used to store the data. Importantly, rules that were generated previously can be reused by other data partners that have similar data structures or for subsequent updates to the data, rather than starting from scratch. At the time of writing, the CaRROT-Mapper supports transformation to the Person, Observation, Condition Occurrence, Measurement, and Drug Exposure tables.

The conversion and destination tables are captured as “mapping rules” in a single JSON file, which is sent to the data partner.

##### Extract, Transform, and Load Pipeline

The mapping rules developed are used by the Python Extract, Transform, and Load (ETL) pipeline (CaRROT-CDM) [38,41], to convert the data from its native CSV format into the OMOP CDM. The ETL pipeline can be scheduled to run either on demand or whenever new data are available.

#### Federated Querying

The BC|RQUEST query portal (Component E) was licensed as a white-labeled instance from BC Platforms [26-28] and integrated into the Gateway as the Cohort Discovery Search Tool [42]. This component provides an interface allowing approved users (bona fide researchers) to create the definition of their cohort (cohort queries) via a drag and drop interface of available OMOP concepts. Cohort queries (also known as study feasibility queries) are created within the query portal and queued to be collected every few seconds by the BC Platforms BC|LINK software installed (Component D) within the data partners’ Federated Nodes. BC|LINK executes the queries and returns the aggregated results to the query portal.

A single BC|LINK instance can interact with multiple OMOP data sets held by each data partner, and allows each data partner to independently set all data disclosure rules, including data rounding, low number suppression, and whether metadata analysis can be performed. This allows each data partner to determine risk and set appropriate controls as required for each data set rather than a single setting for all data sets. Although the data are stored in software from BC Platforms, they have no mechanism to access the data. All access to the data remains strictly under the control of the data partner.

##### Feasibility Questions

The system allows researchers to dynamically and in real time define the cohorts of interest [42]. They will receive responses from across the network usually within a minute. Such an approach allows the feasibility of potential studies to be understood based on the actual data available and without intervention from data partners. This important feature ensures that researchers understand what is feasible in near real time, while always ensuring the disclosure controls are applied by each data partner.

##### Meta-Analysis

**Figure 2:**
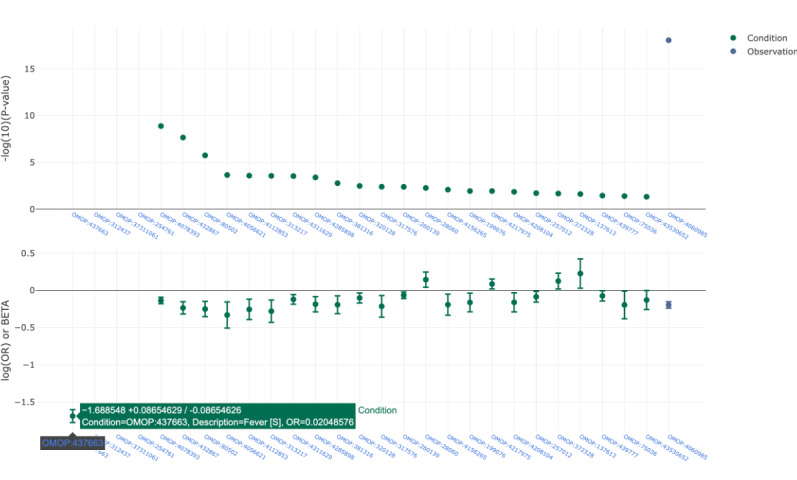
An example phenome-wide association study plot across 4 test data sets comparing females with pneumonia against a background population of female-only samples. The most overrepresented classes include fever (OMOP:437663), disease caused by 2019-nCoV (OMOP:37311061), dysphenia (OMOP:312437), and cough (OMOP:254761). OMOP: Observational Medical Outcomes Partnership.

The capability to perform meta-analysis queries across their data sets is configured by the data partners through an “opt-in” mechanism. Researchers are able to request predefined analyses, through a common user interface, to run across the “opted-in” data sets. An example of a meta-analysis query is to undertake a phenome-wide association study (PheWAS) analysis to understand what phenotypes are linked to different levels of antibody response. In the out-of-the-box capability from BC Platforms, the PheWAS analysis is initially treated as 2 availability queries, one for the case and the other for the control section of the selected cohorts. The subsets of individuals returning within each availability query are then selected from the database, and a PheWAS/Forest analysis is performed across the OMOP CDM search space. This identifies the most overrepresented and underrepresented terms within each cohort. The output is returned to BC|RQUEST as an array of data, which is combined with the information from other cohorts to find the common “META” terms that are overrepresented and underrepresented across all the cohorts. This information is displayed back to the user in the form of a PheWAS plot or a forest plot, or downloaded as a Boolean table of the results. An example is shown in Figure 2.

Custom meta-analytic modules can also be implemented within the BC Platforms ecosystem. These can be developed in either R or Python. Work to develop more advanced statistical meta-analysis and investigations into potential biases or statistical challenges will form future research.

##### Data Access Requests

The data discovery and meta-analysis tools only report aggregated-level data. Details of the data sources queried are provided, so that when an appropriate cohort is identified, direct contact with the appropriate data partner can be made to initiate data governance approvals for a specific research study, which requires individual-level data analysis using the cohort identified. The Gateway-standardized governance application process (Five Safes [safe projects, safe people, safe settings, safe outputs, and safe data] [43,44] form) can be used to streamline the effort required to obtain approvals from data partners who have adopted the standard [45].

### Engagement With Patients and the Public

We have patient and public representatives co-leading the project, with 2 lay member co-investigators and a public and patient group. Representatives attend our work package, leadership team, and advisory board meetings. Representatives reviewed all the controls developed for CO-CONNECT, ensuring we are protecting patient confidentiality and maintaining trust. We developed a series of public-facing videos: Overview [35], Finding Data [46], and Analyzing Data [47]. We also drafted a lay summary and Frequently Asked Questions page [48].

### Ethics Considerations

Research ethics approval was not required for this project as each data partner maintains their own governance and ethics for the original research studies. Anyone requiring access to the platform to perform research needs to apply for their own ethics approval.

## Results

### Data Coverage

The CO-CONNECT consortium includes 41 leaders from 29 different organizations across the 4 devolved UK Nations and is currently on-boarding over 56 different data sets into the platform. The project was launched in October 2020, with 18 months of funding and extension for another 6 months.

CO-CONNECT is focused on the following 3 different types of data partners: (1) COVID-19 research consented cohorts collecting serology data; (2) routinely collected unconsented data from across the United Kingdom; and (3) research cohorts collected prior and during the current pandemic, which CO-CONNECT is enhancing with the ability to link to COVID-19 data (augmented cohorts).

The sources for each type are shown in Table 1. Approximately half of the COVID-19 research cohorts being collected are from health care workers.

**Table 1:** List of data sources incorporated into CO-CONNECT.

Cohort type		Source
**COVID-19 serology cohorts**		
	Health care workers	Co-STARS^a^ [49], COVIDsortium [50], MATCH [51], Oxford Healthcare Workers [52], PANTHER^b^ [53], and SIREN^c^ [54]
	Blood donors	TRACK-COVID [55]
	Care homes	VIVALDI [56]
	Hospitalized patients	ISARIC^d^ [57]
	Schools	sKIDs^e^ [58]
	Education	ACE^f^ [59]
	Random sample of the population of adults registered with a general practitioner in England	REACT-2^g^ [60]
	Hospitalized and community follow-up	FOLLOW-COVID^h^ [61]
**Augmented cohorts**		
	Longitudinal cohorts	ATLAS^i^ [62] (ALSPAC^j^ [63], Generation Scotland [64], GASP^k^ [65], NIHR-BioResource [66], TWINS-UK [67]), and Wellcome Longitudinal Population Study [68] (6 cohorts)
	Respiratory cohorts	HDR^l^ UK BREATHE Hub [69] (17 cohorts)
**Routinely collected health data sources/Trusted Research Environments**		
	England	National Health Service (NHS)–Digital [70] and UK Health and Security Agency (previously Public Health England) [71]
	Scotland	Public Health Scotland (PHS) [72]
	Northern Ireland	HSC^m^ Business Services Organisation [73] and HSC Public Health Agency [74]
	Wales	Secure Anonymised Information Linkage (SAIL) service [75]
	UK-wide	Office of National Statistics (ONS) [76]

^a^Co-STARS: COVID-19 Staff Testing of Antibody Responses Study.

^b^PANTHER: Pandemic Tracking of Healthcare Workers.

^c^SIREN: SARS-CoV-2 Immunity and Reinfection Evaluation Network.

^d^ISARIC: International Severe Acute Respiratory and emerging Infections Consortium.

^e^sKIDS: COVID-19 Surveillance in School Kids.

^f^ACE: Asymptomatic COVID-19 in Education.

^g^REACT-2: Real-time Assessment of Community Transmission 2.

^h^FOLLOW-COVID: Focused Longitudinal Observational Study to Improve Knowledge of COVID-19.

^i^ATLAS: Access Points to Tissue, Longitudinal Data, Archives, and Samples.

^j^ALSPAC: Avon Longitudinal Study of Parents And Children.

^k^GASP: Genetics of Asthma Severity and Phenotypes.

^l^HDR: Health Data Research.

^m^HSC: Health and Safety Commission.

### Data Sets Onboarded

The HDR UK Cohort Discovery Service was first launched in April 2021. At the time of writing, the following data partners are live within the HDR Cohort Discovery Tool: ALSPAC (Avon Longitudinal Study of Parents And Children), PANTHER (Pandemic Tracking of Healthcare Workers), GASP (Genetics of Asthma Severity and Phenotypes), ACE (Asymptomatic COVID-19 in Education) Cohort, MATCH, Generation Scotland, NIHR Bioresource, FOLLOW-COVID (Focused Longitudinal Observational Study to Improve Knowledge of COVID-19), Co-STARS (COVID-19 Staff Testing of Antibody Responses Study), TRACK-COVID, and COVIDSortium. The following data partners have governance approvals in place and are in the process of being on-boarded: ISARIC4C (International Severe Acute Respiratory and emerging Infections Consortium), UKHSA (SIREN [SARS-CoV-2 Immunity and Reinfection Evaluation Network] and sKids [COVID-19 Surveillance in School Kids]), REACT-1 (Real-time Assessment of Community Transmission 1), REACT-2 (Real-time Assessment of Community Transmission 2), Oxford Healthcare Workers, TWINS-UK, Wales/SAIL (COVID Vaccination Dataset [CVVD] and COVID Test Results [PATD]), Public Health Scotland (13 different data sets), and Northern Ireland (COVID antigen testing pillar 1 and 2, COVID-19 Vaccination, Admissions, and Discharges, Emergency Department). CO-CONNECT is currently working with the remaining data partners to obtain relevant governance approvals for their data sets to be incorporated into the platform.

This is an innovative infrastructure project to support research at scale across the United Kingdom. The unique nature of the project made it challenging to onboard data sets from different organizations in terms of (1) different data governance processes with varying information required, (2) different levels of understanding of governance requirements and the technical solution, and (3) delays in governance due to capacity during a pandemic. To overcome these challenges, approaches, such as one-to-one sessions, technical guidance workshops, and sharing a governance guidance pack [77] with data partners, were used. We also commissioned explainer videos to explain the system and how it protects patient confidentiality for both data partners and the general public [35,46,47]. We plan on describing these challenges and lessons learnt elsewhere.

### User Feedback

HDR UK undertook market research in December 2021 and January 2022 led by an external agency. The research included audience mapping, analysis, and 30 interviews with health data users from a range of sectors, including industry, academia, and the National Health Service (NHS). Overall, Cohort Discovery was very positively received, and a short-term goal now for HDR is to “build on perceived successes in search functionality, that is, the Cohort Discovery Tool.” The feedback from users was that the Cohort Discovery Tool could help address some of the needs around metadata and that the approach reflected the way in which many want to understand, assess, and access data. The users recognized the value of standardization across data collection/data terms to vastly increase the options for linking and comparing data and wanted to see the tool developed further. There are currently 150 active users. We expect this to increase with additional data sets live on the system and promotion of the resource.

### Key Outcome of the CO-CONNECT Infrastructure

**Figure 3:**
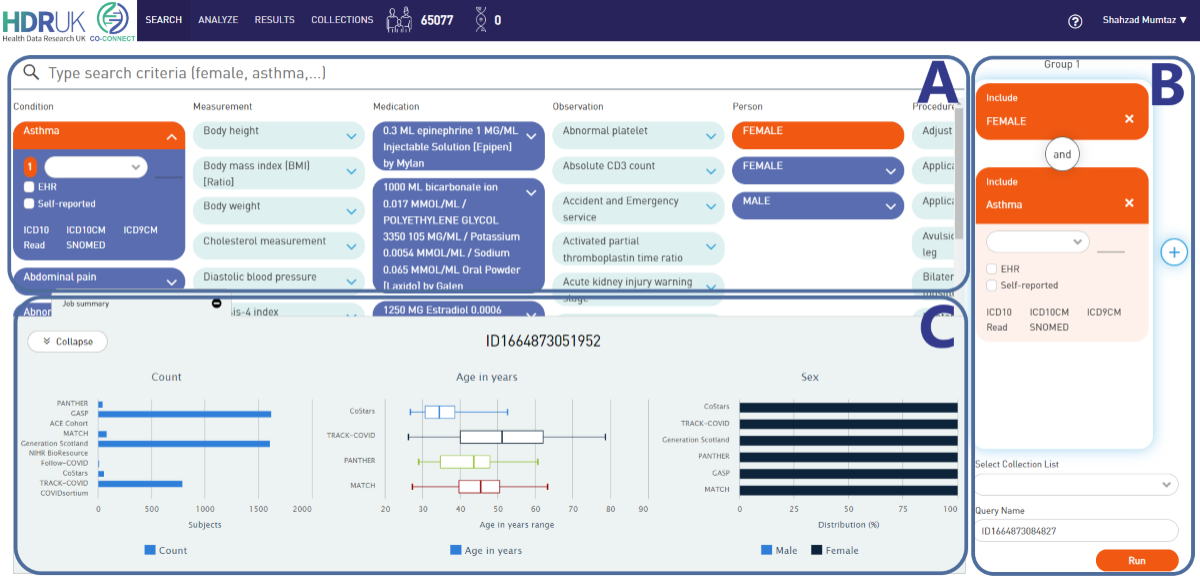
The HDR UK Cohort Discovery Tool. The interface enables the user to define their cohort search criteria and displays aggregate results across different data sets. The available cohort search criteria (A) are used to create selected cohort criteria (a drag and drop feature, B). Results matching the cohort search criteria across different data sets are presented in the output once the federated queries are completed (C). HDR: Health Data Research.

CO-CONNECT is enabling rapid data discovery of data sets available from each data partner via near instantaneous aggregate-level cohort building queries. Figure 3 shows the Cohort Discovery Tool, available from the Gateway, with an example query of “all females with asthma” against all available data sets. The aggregated results presented in the Figure 3 example include overall counts, and age and gender distributions across all data partners down to the individual data set level, enabling researchers to rapidly refine their cohorts of interest.

**Figure 4:**
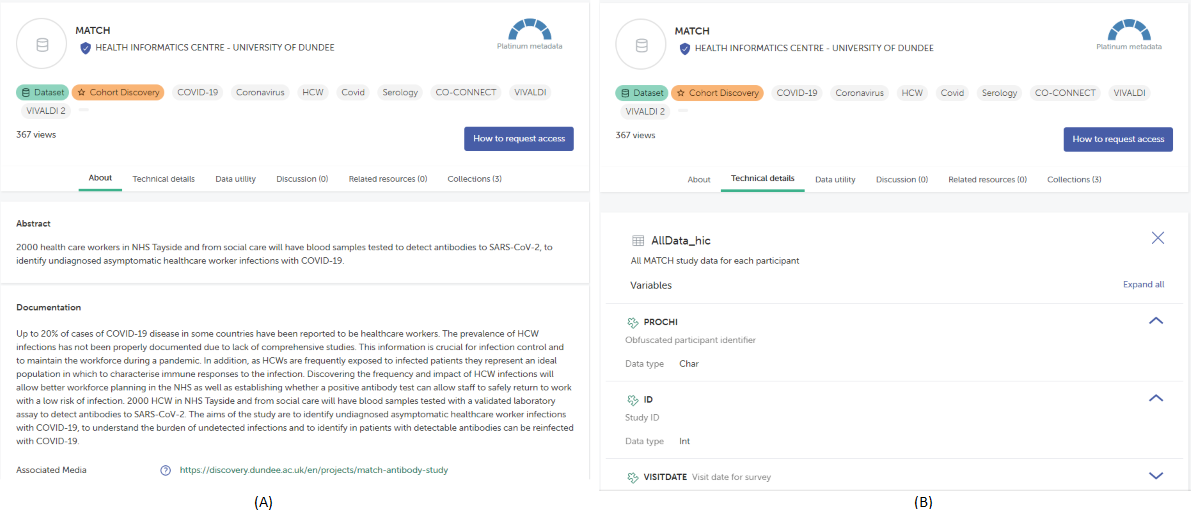
An example of the static metadata found in the data catalogue of the HDR Innovation Gateway (MATCH data set). (A) Summary of the MATCH data set. (B) Technical details – a list of tables with their field names and data types. HDR: Health Data Research.

Prior to the Cohort Discovery Tool being embedded within the Gateway, the only information a researcher could access was a static metadata catalogue of data sets/cohorts, such as overall population size, table names, and field names with their data types and descriptions, as shown in Figure 4. In contrast, the Cohort Discovery Tool enables researchers to dynamically define a cohort search query and get aggregate counts matching the cohort search criteria for the data sets.

CO-CONNECT allows meta-analysis across the data sets, such as time series or binary comparisons. When researchers and public health groups need access to individual-level pseudonymized data for detailed analysis (over and above aggregate-level analysis available in the tool), the data for the analysis can be moved to a TRE for access by the researchers. The CO-CONNECT architecture is being enhanced to support semiautomated streamlined extracts of standardized linked data from across multiple data partners for access within a TRE [12].

### Future Work

We are working with data partners to research mechanisms in which, where practical to do so, global pseudoidentifiers are identical across different data partners. This would be achieved by the use of a common one-way irreversible cryptographic hashing algorithm applied to identifiers, such as NHS and Community Health Index numbers, and would enable data linkage across data partners. These global pseudoidentifiers are never shared outside of the group of data partners. This would enable data linkage across data sets from different data sources (see section on extraction into a TRE below) and would support duplicate detection.

To support duplicate detection for the aggregate-level data discovery and meta-analysis functionality, we have a minimum viable product developed with BC Platforms ensuring that for each query, the global pseudoidentifiers are replaced by query-specific identifiers within the VM. The list of query-specific identifiers is returned along with the aggregate-level counts associated with the query to a secure temporary location, and the IDs from each data partner can then be automatically compared, providing the user who initiated the query with an estimate of the overlap of individuals across different cohorts. For example, 200 people met the search criteria from data partner A, while 350 people met the search criteria form data partner B, and 27 people were the same individuals from data partners A and B. The query-specific identifiers are never made visible to the user and are generated afresh using a new salt (random data that is used as an additional input to a one-way function that hashes data) for each query before being deleted at the query end. CO-CONNECT is working across data partners to assess the feasibility of enabling such functionality.

### Extraction Into a TRE

The CO-CONNECT architecture is being enhanced to support the linkage and extraction of individual-level data from the pseudonymized databases within each data partner into a TRE. There are many TREs operating across the United Kingdom, such as the National TREs for England [70], Scotland [72], Northern Ireland [74], and Wales [75]. These example TREs were also data partners of CO-CONNECT. Data partners can choose whether to use the CO-CONNECT semiautomated pipeline or their own in-house methods for data extraction. When extracting research project–specific individual-level data into a TRE, the global pseudoidentifiers will be replaced with new project-specific pseudoidentifiers prior to export. This means that data from different data partners are linkable by the research group within the TRE for the specific research project without the global identifiers being shared. As the pseudoidentifiers are project specific, linkage across different research projects is safeguarded against.

## Discussion

### Hybrid Infrastructure

We have brought together EHR data of national importance into a federated platform. The data can be queried via the Cohort Discovery Tool in the HDR UK Innovation Gateway. An open-source set of tools were developed to standardize the mapping of data into the OMOP standard without the need to view the individual-level data.

CO-CONNECT evolved from a recognized need across multiple domains for a transformative step in the ability for researchers to discover data across a range of data assets. Centralized data architectures have historically been used when it is possible to set up flow of data to a single location, under a single set of governance approvals (such as national registries) and usually from a small number of organizations. This has been very effective in the United Kingdom with flow of data from the NHS bodies to respective national data repositories, especially when there is a legal mandate, such as the registration of a disease. Such approaches are successful at supporting certain research activities, such as epidemiology, where evaluating the prevalence of a disease can be undertaken with relative ease.

Centralized infrastructure brings economy of scale and the ability to have a specialized team of technologists that can bring standardization to the process and policy. However, such centralized infrastructure cannot infinitely scale to accommodate all data that might be required to perform analyses. It is also clear that while certain aspects of epidemiological research can be undertaken via a centralized model, such as the prevalence or risk associated with different demographic characteristics, it is likely there will never be enough data held in a single location to help answer questions of causation rather than retrospective observations. There is a need to combine information from multiple sources to increase power and generalizability. Aside from technical constraints, the public are equally uncomfortable with their sensitive data being shared widely or within a central database, and thus, keeping all individual-level data local improves patient trust [78,79].

COVID-19 brought a set of challenges such that data analysis and infrastructure were required across and between the national centralized databases of the 4 nations of the United Kingdom. CO-CONNECT was tasked to deliver an overarching platform across existing centralized infrastructure, as well as cater to academic collection of data. This was not a simple distinction between federated or centralized models, but a hybrid infrastructure to support both federation across national centralized TREs and inclusion of specific research data sets into a single ecosystem of collaboration and co-existence.

### Federated Cohort Discovery

CO-CONNECT has been designed to work for the whole population of the United Kingdom. These data come from many databases with thousands of fields held within each of the 4 nations. The technical novelty of the architecture lies in the fact that it supports reproducible and semiautomated processing/tooling for inclusion of new data sets and addition of new fields without significant additional effort compared with OHDSI’s tooling available [80]. Therefore, while federated cohort discovery tools do exist, this is the first time such a system has been designed to be deployed at this scale. The CO-CONNECT approach federates cohort discovery from one simple-to-use application. It will enable the querying of data sets from the 4 nations within the United Kingdom without separate data governance applications. Researchers are able to query data sets immediately and interactively as part of their feasibility study without the substantial overhead of contacting each data partner to ask about running multiple bespoke feasibility queries.

### Centralized Data Curation

All source data are transformed into the OMOP data model via our teams in Dundee, Nottingham, and Edinburgh. The software developed allows the maps to be created centrally but applied locally by each data partner. This retains a clear separation for data governance and importantly enables data partners to be included with minimum effort for them. This is performed via reproducible code, which ensures transformations to the data from the source to the new model are consistent across projects. The mapping of the data into OMOP is supported by the core data science team across all the data partners, ensuring standardization in mapping. Using a reproducible workflow works in concert with automation to support the regular updating of data across the platform via a consistent ETL mechanism.

### Data Extraction

Federated analytics is emerging as a credible alternative, but it was recognized that certain analyses cannot be undertaken using current federated approaches. Therefore, despite putting in place a federated architecture, we are designing the approach to allow subsets of pseudonymized data for answering a specific research project to be extracted into a single TRE. Data curation to a standard will aid this process significantly, as all data have already been curated to the OMOP CDM. The automation of these steps streamlines the process of transitioning to individual-level data from a higher-level query and reduces costs. The data partners who chose to adopt the automated process will require limited resource to release data, and throughput can scale without additional investment. Researchers will receive data in a familiar format, allowing them to reuse existing methodologies. The data in the original format can also be provided to the researchers should this be required.

### Comparison With Other International COVID-19 Initiatives

We reviewed other existing COVID data efforts across the world [81-86]. Most projects focus on the analysis of data sets that were already known to the researchers, whereas CO-CONNECT (as well as CODEX [84,85]) also provides the capability to search for specific cohorts of data for feasibility analysis across population-wide data.

Projects, such as 4CE [83], N3C [86], and the COVID-19 Data Exchange Platform [84], took a centralized approach. 4CE [83] transformed data into a common format at each data source and then obfuscated the values. 4CE transferred the files to a shared central location, merging the files from different sources so analysis could take place. N3C [86] supported data in 4 different CDMs: PCORnet [87], OMOP, i2b2/ACT [88,89], and TriNetX [27], bringing the data into a central cloud platform for secure analysis. The COVID-19 Data Exchange Platform supported federated nodes in the i2b2 [23] format and federated queries, and also provided a centralized analysis platform. They encountered challenges with obtaining ethical approval for transferring data onto the centralized platform, and at the time of writing, data from only 350 patients had been transferred.

The COVID-19 SCOR project [81] plans to utilize the MedCo software [82], which uses collective homomorphic encryption and obfuscation across decentralized data sources. MedCo is deployable on top of standardized systems, such as i2b2 [23]/SHRINE [90] and TranSMART [91]. The unCoVer project aims to use the DataSHIELD [25] software to perform federated analytics across 18 countries [92]. As far as we are aware, all these federated analytics solutions require inbound connections to the data and opening ports on firewalls. In the case of MedCo, encryption of the data reduces the privacy risks associated with inbound connections to the data.

The approach taken really depends on the attitudes of the data partners. In CO-CONNECT, most partners would not accept inbound connections into their secure environment and would not be happy to place sensitive data in an area where an inbound connection could be allowed, regardless of encryption or access controls. For those reasons, CO-CONNECT was built on the assumption of never requiring an inbound connection to the federated data to either curate the data or run a feasibility analysis and meta-analysis. As an additional level of security, on top of not allowing inbound queries, the CO-CONNECT architecture could adopt homomorphic encryption in the future to support more advanced federated queries where researchers need to see the underpinning data.

CO-CONNECT, unlike other COVID-19 solutions, supports data partners to automate the mapping of their data into a CDM without having to see the underpinning data. This is advantageous as most data partners do not have their data mapped into the OMOP CDM or the technical capability to do so.

### Current Status and Contributions

Metadata covering the data sources are now available to search openly within the Gateway [30]. National and international researchers can request access to the enhanced dynamic cohort discovery capability within the Gateway. Access to individual-level subsets of data by national and international researchers can also be requested via the streamlined governance application process [45].

We welcome requests to onboard data sets into CO-CONNECT; further details are available via the corresponding author.

The platform has been designed to be disease agnostic. COVID-19 has supported the need for such a platform to provide data at pace. However, the model can be reused to support research at pace for other disease areas. The platform underpins the recently funded HDR UK/MRC Alleviate Hub for Pain Research [93], and the architecture and support for cohort building will be supported and enhanced by HDR after the end of CO-CONNECT funding. Exemplar projects using the architecture are planned for the next phase of HDR funding.

### Conclusions

We have introduced the CO-CONNECT federated architecture, which addresses the challenges of fragmentation of data and lack of interoperability and standardization, as well as the challenge of linkage of high value data assets to other data assets providing new scientific insights. The architecture has been designed around the following core principles: (1) maintaining patient confidentiality, trust, and data security; (2) empowering data partners to be interconnected in a sustainable environment; (3) utilization and re-enforcement of TREs to analyze data; (4) a focus on data engineering to ensure technical legacy for wider use; and (5) a standard-based approach to ensure interoperability, repeatability, and connectivity to other initiatives, responding to the most pressing needs of the public health and research communities.

The development of this platform will empower public health organizations, research groups, and industry bodies to answer key questions about the COVID-19 pandemic and its effects on human health in a streamlined timely manner, as has been needed for EHRs for many years [15,21]. The solution enables rapid cohort-building data discovery across data partners. None of the data partners had such capability for researchers prior to CO-CONNECT. CO-CONNECT has simplified the complex task of requesting access to each individual data set, by providing transparency on what data are available and from where, and how to request access if individual-level data analysis is required. CO-CONNECT provides novel real-time functionality compared to static metadata dictionaries and descriptions of cohorts already provided within the Gateway.

The immediate impact of CO-CONNECT is the fast, accessible, and standardized availability of aggregate COVID-19–related data, to inform key public health decisions and help tackle the COVID-19 pandemic at pace. As more data sets are onboarded, this will become more powerful.

## Abbreviations

CaRROT: Convenient and Reusable Rapid Ontology Transformer
CDM: common data model
CO-CONNECT: COVID - Curated and Open Analysis and Research Platform
EHR: electronic health record
ETL: Extract, Transform, and Load
GDPR: General Data Protection Regulations
HDR: Health Data Research
NHS: National Health Service
OHDSI: Observational Health Data Science and Informatics
OMOP: Observational Medical Outcomes Partnership
PheWAS: phenome-wide association study
TRE: Trusted Research Environment
VM: virtual machine

## References

[1] Richter A, Plant T, Kidd M, Bosworth A, Mayhew M, Megram O, Ashworth F, Crawford L, White T, Moles-Garcia E, Mirza J, Percival B, McNally A (2020). How to establish an academic SARS-CoV-2 testing laboratory. Nat Microbiol.

[2] Park S, Elliott J, Berlin A, Hamer-Hunt J, Haines A (2020). Strengthening the UK primary care response to covid-19. BMJ.

[3] (2020). Oral evidence: UK Science, Research and Technology Capability and Influence in Global Disease Outbreaks, HC 136. Science and Technology Committee.

[4] Better, broader, safer: using health data for research and analysis. GOV UK.

[5] Cavallaro F, Lugg-Widger F, Cannings-John R, Harron K (2020). Reducing barriers to data access for research in the public interest—lessons from covid-19. BMJ Opinion.

[6] Taylor JA, Crowe S, Espuny Pujol F, Franklin RC, Feltbower RG, Norman LJ, Doidge J, Gould DW, Pagel C (2021). The road to hell is paved with good intentions: the experience of applying for national data for linkage and suggestions for improvement. BMJ Open.

[7] Macnair A, Love SB, Murray ML, Gilbert DC, Parmar MKB, Denwood T, Carpenter J, Sydes MR, Langley RE, Cafferty FH (2021). Accessing routinely collected health data to improve clinical trials: recent experience of access. Trials.

[8] (2020). The researchers’ experience when attempting to access health data for research. NCRI.

[9] Larrucea X, Moffie M, Asaf S, Santamaria I (2020). Towards a GDPR compliant way to secure European cross border Healthcare Industry 4.0. Computer Standards & Interfaces.

[10] (2020). Data sharing agreement template. NHS.

[11] Lin C, Stephens KA, Baldwin L, Keppel GA, Whitener RJ, Echo-Hawk A, Korngiebel D (2014). Developing Governance for Federated Community-based EHR Data Sharing. AMIA Jt Summits Transl Sci Proc.

[12] Hubbard T, Reilly G, Varma S, Seymour D (2020). Trusted Research Environments (TRE) Green Paper. Zenodo.

[13] Kruse CS, Smith B, Vanderlinden H, Nealand A (2017). Security Techniques for the Electronic Health Records. J Med Syst.

[14] Sloan P (2014). The Compliance Case for Information Governance. Richmond Journal of Law & Technology.

[15] Trifan A, Oliveira JL (2019). Patient data discovery platforms as enablers of biomedical and translational research: A systematic review. J Biomed Inform.

[16] (2020). Sharing Sensitive Health Data in a Federated Data Consortium Model: An Eight-Step Guide. World Economic Forum.

[17] Knoppers BM (2014). Framework for responsible sharing of genomic and health-related data. Hugo J.

[18] Dursi LJ, Bozoky Z, de Borja R, Li H, Bujold D, Lipski A, Rashid SF, Sethi A, Memon N, Naidoo D, Coral-Sasso F, Wong M, Quirion P, Lu Z, Agarwal S, Pavlov Y, Ponomarev A, Husic M, Pace K, Palmer S, Grover SA, Hakgor S, Siu LL, Malkin D, Virtanen C, Pugh TJ, Jacques P, Joly Y, Jones SJ, Bourque G, Brudno M (2021). CanDIG: Federated network across Canada for multi-omic and health data discovery and analysis. Cell Genomics.

[19] CINECA - Common Infrastructure for National Cohorts in Europe, Canada, and Africa.

[20] European Health Data Evidence Network.

[21] Rahimzadeh V, Dyke SO, Knoppers BM (2016). An International Framework for Data Sharing: Moving Forward with the Global Alliance for Genomics and Health. Biopreserv Biobank.

[22] Dobbins NJ, Spital CH, Black RA, Morrison JM, de Veer B, Zampino E, Harrington RD, Britt BD, Stephens KA, Wilcox AB, Tarczy-Hornoch P, Mooney SD (2020). Leaf: an open-source, model-agnostic, data-driven web application for cohort discovery and translational biomedical research. J Am Med Inform Assoc.

[23] Murphy SN, Weber G, Mendis M, Gainer V, Chueh HC, Churchill S, Kohane I (2010). Serving the enterprise and beyond with informatics for integrating biology and the bedside (i2b2). J Am Med Inform Assoc.

[24] Schüttler C, Prokosch H, Hummel M, Lablans M, Kroll B, Engels C, German Biobank Alliance IT Development Team (2021). The journey to establishing an IT-infrastructure within the German Biobank Alliance. PLoS One.

[25] Wolfson M, Wallace SE, Masca N, Rowe G, Sheehan NA, Ferretti V, LaFlamme P, Tobin MD, Macleod J, Little J, Fortier I, Knoppers BM, Burton PR (2010). DataSHIELD: resolving a conflict in contemporary bioscience--performing a pooled analysis of individual-level data without sharing the data. Int J Epidemiol.

[26] BC Platforms.

[27] Stacey J, Mehta M (2017). Using EHR Data Extraction to Streamline the Clinical Trial Process. Clinical Researcher.

[28] Clinerion.

[29] Data science and AI in the age of COVID-19 – report. The Alan Turing Institute.

[30] HDR UK Innovation Gateway.

[31] Sebire NJ, Cake C, Morris AD (2020). HDR UK supporting mobilising computable biomedical knowledge in the UK. BMJ Health Care Inform.

[32] COVID-19 National Core Studies. HDR UK.

[33] Scientific Advisory Group for Emergencies. GOV UK.

[34] Wilkinson MD, Dumontier M, Aalbersberg IJ, Appleton G, Axton M, Baak A, Blomberg N, Boiten J, da Silva Santos LB, Bourne PE, Bouwman J, Brookes AJ, Clark T, Crosas M, Dillo I, Dumon O, Edmunds S, Evelo CT, Finkers R, Gonzalez-Beltran A, Gray AJ, Groth P, Goble C, Grethe JS, Heringa J, 't Hoen PAC, Hooft R, Kuhn T, Kok R, Kok J, Lusher SJ, Martone ME, Mons A, Packer AL, Persson B, Rocca-Serra P, Roos M, van Schaik R, Sansone S, Schultes E, Sengstag T, Slater T, Strawn G, Swertz MA, Thompson M, van der Lei J, van Mulligen E, Velterop J, Waagmeester A, Wittenburg P, Wolstencroft K, Zhao J, Mons B (2016). The FAIR Guiding Principles for scientific data management and stewardship. Sci Data.

[35] CO-CONNECT - Overview. YouTube.

[36] OMOP CDM v5.3. OHDSI GitHub.

[37] Cox S, Macdonald C, Lea D, Adejumo S, Panagi V, Tarr S, Mumtaz S, Santos R, Schlessinger D, Quinlan P (2022). HDRUK/CaRROT-Mapper: 2.0.1. Zenodo.

[38] Macdonald C, Panagi V, Tarr S, Santos R, Schlessinger D, Mumtaz S (2022). HDRUK/CaRROT-CDM: CaRROT CDM Builder Version 0.6.0. Zenodo.

[39] OHDSI - Observational Health Data Sciences and Informatics.

[40] White Rabbit. OHDSI GitHub.

[41] Cox S, Macdonald C, Mumtaz S, Panagi V, Tarr S, Quinlan P CaRROT-Docs. HDRUK GitHub.

[42] Cohort Discovery. HDR UK Innovation Gateway.

[43] Ritchie F (2017). The 'Five Safes': a framework for planning, designing and evaluating data access solutions. Zenodo.

[44] The ‘Five Safes’ – Data Privacy at ONS. Office for National Statistics.

[45] Data Access Request Process Overview. HDR UK Innovation Gateway.

[46] CO-CONNECT - Finding Data. YouTube.

[47] CO-CONNECT - Accessing and Analysing Data. YouTube.

[48] CO-CONNECT | Unleashing the power of data through discovery.

[49] Grandjean L, Saso A, Torres A, Lam T, Hatcher J, Thistlethwayte R, Harris M, Best T, Johnson M, Wagstaffe H, Ralph E, Mai A, Colijn C, Breuer J, Buckland M, Gilmour K, Goldblatt D, Co-Stars Study Team (2020). Humoral Response Dynamics Following Infection with SARS-CoV-2. medRxiv.

[50] Augusto JB, Menacho K, Andiapen M, Bowles R, Burton M, Welch S, Bhuva AN, Seraphim A, Pade C, Joy G, Jensen M, Davies RH, Captur G, Fontana M, Montgomery H, O'Brien B, Hingorani AD, Cutino-Moguel T, Abbass H, Alfarih M, Alldis Z, Baca GL, Boulter A, Bracken OV, Bullock N, Champion N, Chan C, Couto-Parada X, Dieobi-Anene K, Feehan K, Figtree G, Figtree MC, Finlay M, Forooghi N, Gibbons JM, Griffiths P, Hamblin M, Howes L, Itua I, Jones M, Jardim V, Kapil V, Jason Lee W, Mandadapu V, Mfuko C, Mitchelmore O, Palma S, Patel K, Petersen SE, Piniera B, Raine R, Rapala A, Richards A, Sambile G, Couto de Sousa J, Sugimoto M, Thornton GD, Artico J, Zahedi D, Parker R, Robathan M, Hickling LM, Ntusi N, Semper A, Brooks T, Jones J, Tucker A, Veerapen J, Vijayakumar M, Wodehouse T, Wynne L, Treibel TA, Noursadeghi M, Manisty C, Moon JC, McKnight (2020). Healthcare Workers Bioresource: Study outline and baseline characteristics of a prospective healthcare worker cohort to study immune protection and pathogenesis in COVID-19. Wellcome Open Res.

[51] Abo-Leyah H, Gallant S, Cassidy D, Giam Y, Killick J, Marshall B, Hay G, Snowdon C, Hothersall EJ, Pembridge T, Strachan R, Gallant N, Parcell BJ, George J, Furrie E, Chalmers JD (2021). The protective effect of SARS-CoV-2 antibodies in Scottish healthcare workers. ERJ Open Res.

[52] Lumley SF, O'Donnell D, Stoesser NE, Matthews PC, Howarth A, Hatch SB, Marsden BD, Cox S, James T, Warren F, Peck LJ, Ritter TG, de Toledo Z, Warren L, Axten D, Cornall RJ, Jones EY, Stuart DI, Screaton G, Ebner D, Hoosdally S, Chand M, Crook DW, O'Donnell AM, Conlon CP, Pouwels KB, Walker AS, Peto TE, Hopkins S, Walker TM, Jeffery K, Eyre DW, Oxford University Hospitals Staff Testing Group (2021). Antibody Status and Incidence of SARS-CoV-2 Infection in Health Care Workers. N Engl J Med.

[53] Valdes AM, Moon JC, Vijay A, Chaturvedi N, Norrish A, Ikram A, Craxford S, Cusin LM, Nightingale J, Semper A, Brooks T, McKnight A, Kurdi H, Menni C, Tighe P, Noursadeghi M, Aithal G, Treibel TA, Ollivere BJ, Manisty C (2021). Longitudinal assessment of symptoms and risk of SARS-CoV-2 infection in healthcare workers across 5 hospitals to understand ethnic differences in infection risk. EClinicalMedicine.

[54] Hall VJ, Foulkes S, Charlett A, Atti A, Monk EJM, Simmons R, Wellington E, Cole MJ, Saei A, Oguti B, Munro K, Wallace S, Kirwan PD, Shrotri M, Vusirikala A, Rokadiya S, Kall M, Zambon M, Ramsay M, Brooks T, Brown CS, Chand MA, Hopkins S, SIREN Study Group (2021). SARS-CoV-2 infection rates of antibody-positive compared with antibody-negative health-care workers in England: a large, multicentre, prospective cohort study (SIREN). Lancet.

[55] The TRACK-COVID Study. TRACK-COVID.

[56] VIVALDI Study. UCL.

[57] Docherty AB, Harrison EM, Green CA, Hardwick HE, Pius R, Norman L, Holden KA, Read JM, Dondelinger F, Carson G, Merson L, Lee J, Plotkin D, Sigfrid L, Halpin S, Jackson C, Gamble C, Horby PW, Nguyen-Van-Tam JS, Ho A, Russell CD, Dunning J, Openshaw PJ, Baillie JK, Semple MG, ISARIC4C investigators (2020). Features of 20 133 UK patients in hospital with covid-19 using the ISARIC WHO Clinical Characterisation Protocol: prospective observational cohort study. BMJ.

[58] COVID-19 surveillance in school KIDs (sKIDs): pre and primary schools. GOV UK.

[59] Asymptomatic COVID-19 in Education (ACE) Cohort. HDR UK Innovation Gateway.

[60] Real-time Assessment of Community Transmission (REACT) Study. Imperial College London.

[61] FOLLOW-COVID. NHS.

[62] ATLAS: Advanced data search tool for researchers. UKCRC Tissue Directory and Coordination Centre.

[63] Boyd A, Golding J, Macleod J, Lawlor DA, Fraser A, Henderson J, Molloy L, Ness A, Ring S, Davey Smith G (2013). Cohort Profile: the 'children of the 90s'--the index offspring of the Avon Longitudinal Study of Parents and Children. Int J Epidemiol.

[64] Smith B, Campbell A, Linksted P, Fitzpatrick B, Jackson C, Kerr S, Deary I, Macintyre DJ, Campbell H, McGilchrist M, Hocking L, Wisely L, Ford I, Lindsay R, Morton R, Palmer C, Dominiczak A, Porteous D, Morris A (2013). Cohort Profile: Generation Scotland: Scottish Family Health Study (GS:SFHS). The study, its participants and their potential for genetic research on health and illness. Int J Epidemiol.

[65] Shrine N, Portelli MA, John C, Soler Artigas M, Bennett N, Hall R, Lewis J, Henry AP, Billington CK, Ahmad A, Packer RJ, Shaw D, Pogson ZEK, Fogarty A, McKeever TM, Singapuri A, Heaney LG, Mansur AH, Chaudhuri R, Thomson NC, Holloway JW, Lockett GA, Howarth PH, Djukanovic R, Hankinson J, Niven R, Simpson A, Chung KF, Sterk PJ, Blakey JD, Adcock IM, Hu S, Guo Y, Obeidat M, Sin DD, van den Berge M, Nickle DC, Bossé Y, Tobin MD, Hall IP, Brightling CE, Wain LV, Sayers I (2019). Moderate-to-severe asthma in individuals of European ancestry: a genome-wide association study. Lancet Respir Med.

[66] NIHR BioResource.

[67] Moayyeri A, Hammond CJ, Valdes AM, Spector TD (2013). Cohort Profile: TwinsUK and healthy ageing twin study. Int J Epidemiol.

[68] Cohorts - COVID-19 Longitudinal Health and Wellbeing National Core Study. UCL.

[69] BREATHE - Health Data Research Hub.

[70] Data dashboards. NHS.

[71] Public Health England. GOV UK.

[72] COVID-19. Public Health Scotland.

[73] Business Services Organisation.

[74] Public Health Agency.

[75] SAIL Databank - The Secure Anonymised Information Linkage Databank.

[76] Coronavirus (COVID-19). Office for National Statistics.

[77] Milligan G, Johnston J, Horban S, Masood E, Cox S, Urwin E, Hadfield A, Giles T, Best J, Macdonald C, Mumtaz S, Beggs J, Chuter A, Quinlan P, Jefferson E (2022). COVID - Curated and OpeN aNalysis and rEsearCh plaTform (CO-CONNECT) Implementation Guide. Zenodo.

[78] Vezyridis P, Timmons S (2017). Understanding the care.data conundrum: New information flows for economic growth. Big Data & Society.

[79] Church J (2021). GP Data for Planning and Research: Letter from Parliamentary Under Secretary of State for Health and Social Care to general practices in England. NHS Digital.

[80] Chapter 6 Extract Transform Load - The Book of OHDSI. OHDSI GitHub.

[81] Raisaro JL, Marino F, Troncoso-Pastoriza J, Beau-Lejdstrom R, Bellazzi R, Murphy R, Bernstam EV, Wang H, Bucalo M, Chen Y, Gottlieb A, Harmanci A, Kim M, Kim Y, Klann J, Klersy C, Malin BA, Méan M, Prasser F, Scudeller L, Torkamani A, Vaucher J, Puppala M, Wong STC, Frenkel-Morgenstern M, Xu H, Musa BM, Habib AG, Cohen T, Wilcox A, Salihu HM, Sofia H, Jiang X, Hubaux JP (2020). SCOR: A secure international informatics infrastructure to investigate COVID-19. J Am Med Inform Assoc.

[82] Raisaro JL, Troncoso-Pastoriza JR, Misbach M, Sousa JS, Pradervand S, Missiaglia E, Michielin O, Ford B, Hubaux J (2019). MedCo: Enabling Secure and Privacy-Preserving Exploration of Distributed Clinical and Genomic Data. IEEE/ACM Trans Comput Biol Bioinform.

[83] Brat GA, Weber GM, Gehlenborg N, Avillach P, Palmer NP, Chiovato L, Cimino J, Waitman LR, Omenn GS, Malovini A, Moore JH, Beaulieu-Jones BK, Tibollo V, Murphy SN, Yi SL, Keller MS, Bellazzi R, Hanauer DA, Serret-Larmande A, Gutierrez-Sacristan A, Holmes JJ, Bell DS, Mandl KD, Follett RW, Klann JG, Murad DA, Scudeller L, Bucalo M, Kirchoff K, Craig J, Obeid J, Jouhet V, Griffier R, Cossin S, Moal B, Patel LP, Bellasi A, Prokosch HU, Kraska D, Sliz P, Tan ALM, Ngiam KY, Zambelli A, Mowery DL, Schiver E, Devkota B, Bradford RL, Daniar M, Daniel C, Benoit V, Bey R, Paris N, Serre P, Orlova N, Dubiel J, Hilka M, Jannot AS, Breant S, Leblanc J, Griffon N, Burgun A, Bernaux M, Sandrin A, Salamanca E, Cormont S, Ganslandt T, Gradinger T, Champ J, Boeker M, Martel P, Esteve L, Gramfort A, Grisel O, Leprovost D, Moreau T, Varoquaux G, Vie J, Wassermann D, Mensch A, Caucheteux C, Haverkamp C, Lemaitre G, Bosari S, Krantz ID, South A, Cai T, Kohane IS (2020). International electronic health record-derived COVID-19 clinical course profiles: the 4CE consortium. NPJ Digit Med.

[84] Prokosch H, Bahls T, Bialke M, Eils J, Fegeler C, Gruendner J, Haarbrandt B, Hampf C, Hoffmann W, Hund H, Kampf M, Kapsner LA, Kasprzak P, Kohlbacher O, Krefting D, Mang JM, Marschollek M, Mate S, Müller A, Prasser F, Sass J, Semler S, Stenzhorn H, Thun S, Zenker S, Eils R (2022). The COVID-19 Data Exchange Platform of the German University Medicine. Stud Health Technol Inform.

[85] Sedlmayr B, Sedlmayr M, Kroll B, Prokosch H, Gruendner J, Schüttler C (2022). Improving COVID-19 Research of University Hospitals in Germany: Formative Usability Evaluation of the CODEX Feasibility Portal. Appl Clin Inform.

[86] Haendel MA, Chute CG, Bennett TD, Eichmann DA, Guinney J, Kibbe WA, Payne PRO, Pfaff ER, Robinson PN, Saltz JH, Spratt H, Suver C, Wilbanks J, Wilcox AB, Williams AE, Wu C, Blacketer C, Bradford RL, Cimino JJ, Clark M, Colmenares EW, Francis PA, Gabriel D, Graves A, Hemadri R, Hong SS, Hripscak G, Jiao D, Klann JG, Kostka K, Lee AM, Lehmann HP, Lingrey L, Miller RT, Morris M, Murphy SN, Natarajan K, Palchuk MB, Sheikh U, Solbrig H, Visweswaran S, Walden A, Walters KM, Weber GM, Zhang XT, Zhu RL, Amor B, Girvin AT, Manna A, Qureshi N, Kurilla MG, Michael SG, Portilla LM, Rutter JL, Austin CP, Gersing KR, N3C Consortium (2021). The National COVID Cohort Collaborative (N3C): Rationale, design, infrastructure, and deployment. J Am Med Inform Assoc.

[87] Fleurence RL, Curtis LH, Califf RM, Platt R, Selby JV, Brown JS (2014). Launching PCORnet, a national patient-centered clinical research network. J Am Med Inform Assoc.

[88] Visweswaran S, Becich M, D'Itri VS, Sendro E, MacFadden D, Anderson N, Allen K, Ranganathan D, Murphy S, Morrato E, Pincus H, Toto R, Firestein G, Nadler L, Reis S (2018). Accrual to Clinical Trials (ACT): A Clinical and Translational Science Award Consortium Network. JAMIA Open.

[89] Visweswaran S, Samayamuthu MJ, Morris M, Weber GM, MacFadden D, Trevvett P, Klann JG, Gainer V, Benoit B, Murphy SN, Patel L, Mirkovic N, Borovskiy Y, Johnson RD, Wyatt MC, Wang AY, Follett RW, Chau N, Zhu W, Abajian M, Chuang A, Bahroos N, Reeder P, Xie D, Cai J, Sendro ER, Toto RD, Firestein GS, Nadler LM, Reis SE (2021). Development of a COVID-19 Application Ontology for the ACT Network. medRxiv.

[90] Weber GM, Murphy SN, McMurry AJ, MacFadden D, Nigrin DJ, Churchill S, Kohane IS (2009). The Shared Health Research Information Network (SHRINE): A Prototype Federated Query Tool for Clinical Data Repositories. Journal of the American Medical Informatics Association.

[91] Athey BD, Braxenthaler M, Haas M, Guo Y (2013). tranSMART: An Open Source and Community-Driven Informatics and Data Sharing Platform for Clinical and Translational Research. AMIA Jt Summits Transl Sci Proc.

[92] Peñalvo JL, Mertens E, Ademović E, Akgun S, Baltazar AL, Buonfrate D, Čoklo M, Devleesschauwer B, Diaz Valencia PA, Fernandes JC, Gómez EJ, Hynds P, Kabir Z, Klein J, Kostoulas P, Llanos Jiménez L, Lotrean LM, Majdan M, Menasalvas E, Nguewa P, Oh I, O'Sullivan G, Pereira DM, Reina Ortiz M, Riva S, Soriano G, Soriano JB, Spilki F, Tamang ME, Trofor AC, Vaillant M, Van Ierssel S, Vuković J, Castellano JM, unCoVer Network (2021). Unravelling data for rapid evidence-based response to COVID-19: a summary of the unCoVer protocol. BMJ Open.

[93] Alleviate – our Advanced Pain Discovery Platform (APDP) Hub. HDR UK.

